# Updated threshold, renewed problems: should the diagnostic criteria of polycythemia vera be reconsidered? A retrospective cross-sectional cohort study

**DOI:** 10.1590/1806-9282.20230497

**Published:** 2024-03-15

**Authors:** Anıl Uçan, Müfide Okay Özgeyik

**Affiliations:** 1 Eskisehir City Hospital, Department of Internal Medicine – Eskişehir, Turkey.; 2 Eskisehir City Hospital, Department of Hematology – Eskişehir, Turkey.

**Keywords:** Polycythemia, Diagnosis, Hematologic tests, Hematocrit, Hemoglobins

## Abstract

**OBJECTIVE:**

This aim of this study was to evaluate hemoglobin and hematocrit values of polycythemia vera and secondary polycythemia patients with updated World Health Organization thresholds. In addition, by determining our own threshold values, we aimed to demonstrate the necessity of bone marrow biopsy and genetic analysis to be used for further diagnosis in patients with high-normal hematocrit and hemoglobin values.

**METHODS:**

A cross-sectional and retrospective study was performed with the medical records of patients from Eskisehir City Hospital hematology clinics and outpatient clinics between July 1, 2019 and July 1, 2020. The study included patients with polycythemia, divided into two groups according to polycythemia vera and secondary polycythemia. A bone marrow biopsy was performed on patients with either Janus kinase mutation positivity and/or subnormal erythropoietin levels. Receiver operating characteristics analysis was used to find threshold values, and the diagnostic efficiency of these values in differentiating World Health Organization thresholds in 2008 and 2016 was evaluated.

**RESULTS:**

A total of 73 patients were included. The median age was 43.5 years (min: 18; max: 84). The hematocrit value of 54.1 was predicted to diagnose polycythemia vera with a sensitivity of 45% and a specificity of 80%. Subsequent analysis revealed that an hemoglobin value of 17.7 was indicative of diagnosing polycythemia vera with a sensitivity of 60% and a specificity of 63%. The mean follow-up length was 6.4 months (2–12).

**CONCLUSION:**

Our study demonstrated that modified World Health Organization criteria might lead to unnecessary additional tests for polycythemia vera patients with high-normal hemoglobin and hematocrit values.

## INTRODUCTION

Polycythemia vera (PV) is a member of the Philadelphia chromosome-negative (Ph-) chronic myeloproliferative neoplasm diseases^1^. The incidence of the disease is 0.01–2.61 per 100,000 people per year, and the mean age at diagnosis is 60 years^1,2^. Like all myeloproliferative neoplasms (MPNs), PV could transform into acute or chronic myeloid-based myeloid leukemia or neoplasms (yearly incidence of transformation: 0.38% for PV, 0.37% for ET)^3,4^. Secondary acute myeloid leukemias (sAML) were associated with an increased risk of high mortality and a worse prognosis^3,5^. Therefore, the diagnosis of PV plays a critical role in maintaining disease mortality. The current treatment goals in PV are to prevent thromboembolic complications and relieve symptoms. While achieving these goals definitely improves quality of life and survival rates, the existing medical therapies cannot stop PV from developing into leukemia or neoplasms.

However, the condition that should be excluded in the differential diagnosis at the very beginning of the diagnostic algorithm is the presence of secondary polycythemia (SP). It is observed that SP is often caused by tissue hypoxia and rarely by neoplasms that secrete erythropoietin (EPO)^6,7^, and is frequently associated with smoking and chronic obstructive pulmonary disease (COPD). It should be highlighted that, unlike primary polycythemia, the erythroid progenitor lineage of cells does not intrinsically exhibit a deficiency. In both cases of PV and SP, patients usually have nonspecific symptoms on presentation, including fatigue, headache, and dizziness. These similarities have become an obstacle for clinicians to overcome in differential diagnosis for years, and appropriate and cost-effective diagnostic criteria have been tried to be established.

A classification containing updates in the diagnosis of the disease was published by the World Health Organization (WHO) in 2016^8^, and criteria are nowadays frequently used in the diagnosis of MPNs. In the 2016 classification, an update was made, and the first major criteria of high hemoglobin (Hb) (>16.5 g/dL in men or >16.0 g/dL in women) levels were decreased compared to 2008 hematocrit (Hct) (>18.5 g/dL in men or >16.5 g/dL in women)^8^. The suggested revised threshold limits for Hb and Hct, especially for men (men: 13.5–17.5 g/L and Hct: 38.8–50%; women: 12.0–15.5 g/L and Hct: 34.9–44.5%), exhibit a remarkable match with the common reference values found in healthy individuals^1,9^. These patients may be overdiagnosed with PV and examined for serum erythropoietin (EPO) levels and/or Janus kinase 2 (JAK2) mutations without necessity.

As these new thresholds are updated with data from clinico-pathological databases of patients, they have led clinicians to suspect that the impact of the thresholds on disease diagnosis may not be widely applicable in the general population with demographic and geographical differences. The debate continues about the strategies for the overdiagnosis of Philadelphia chromosome-negative (Ph-) chronic MPNs.

In this study, we aimed to compare the Hb and Hct levels in PV and SP patients to current WHO standards. Furthermore, by establishing our own threshold values, we wanted to investigate the necessity of bone marrow biopsy and genetic analysis for further diagnosis in patients with high-normal Hct and Hb values.

## METHODS

### Study design and ethical considerations

This cross-sectional retrospective study includes the evaluation of randomly selected 200 patients who applied to Eskisehir City Hospital hematology clinics and outpatient clinics between July 1, 2019 and July 1, 2020. Approval for the study was obtained from the local ethics committee of Eskişehir Osmangazi University (approval number 2020/328) and was carried out in accordance with the Declaration of Helsinki principles and all applicable regulations. Informed consent was waived (a retrospective study).

### Study groups and eligibility criteria

**Figure 1:**
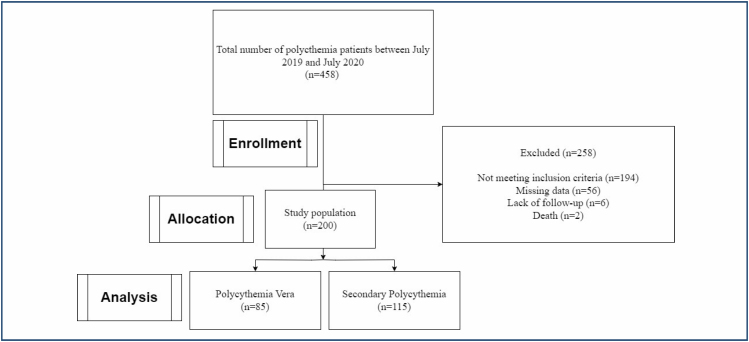
Flowchart of the study.

A total of 458 patients with PV and SP were identified. A flowchart of the study patients is shown in Figure 1. However, out of these subjects, an adequate bone marrow biopsy and complete medical record were available in 85 patients with PV and 115 patients with SP. Patients with PV were diagnosed according to the WHO criteria by two independent hematology specialists^8,10^. However, 11 patients who did not match the WHO criteria for PV at the point of diagnosis or had a mixed diagnosis of myelodysplasia or other myeloproliferative neoplasms such as essential thrombocytosis or primary myelofibrosis were excluded.

The inclusion criteria were being 18 years of age or older and being referred to hematology outpatient clinics with symptoms of polycythemia and hyperviscosity such as erythromelalgia and pruritus. A total of 12 patients diagnosed with essential thrombocythemia were excluded from the study. In patients with no or inadequate data, being pregnant, being under 18 years of age, and using drugs that may cause polycythemia were excluded.

### Data collection

Patient baseline characteristics, comorbidities, smoking status, clinical findings, laboratory results (Hb, Hct, white blood cell (WBC), neutrophil count (Neu), platelet count (Plt), lactate dehydrogenase (LDH), and serum EPO levels), and bone marrow results were obtained from the medical record archives. As a result, we also excluded 56 patients with insufficient data. The mean follow-up length was 6.4 (2–12) months. Six patients who received initial therapy at another hospital were excluded due to a lack of follow-up at our hospital. Two patients were lost to follow-up. In total, follow-up data were available for 200 patients.

All bone marrow biopsies are examined at the Department of Pathology, Eskişehir City Hospital. When all hematologic parameters were within the defined delta limits and no suspected red flags were identified, the CBC was considered normal as follows: WBC: 3–10×10^3^/μL (neutrophils: 1–10×10^3^/μL; platelets: 150–450×10^3^/μL); and no band neutrophil, metamyelocyte, myelocyte, promyelocyte, blast cell, or plasma cells were detectable. The samples were run on a Cell-Dyn Ruby® (Abbott, United States) automated hematology analyzer.

### Statistical analysis

Statistical Package for the Social Sciences (SPSS) was used to conduct the statistical analysis (SPSS, version 22.0, SPSS Inc., Chicago, IL, USA). The distribution of the data was analyzed using the Kolmogorov-Smirnov method. Non-normally distributed data were expressed as a median, interquartile range (IQR), and range. All the continuous data included in this study had a non-normal distribution. Chi-square analysis was used for the comparison of frequencies of the major manifestations and the Student's t-test (Mann-Whitney U test) for the comparison of median values between groups. The ROC analysis was used to determine the threshold values of the numerical parameters that were used to predict disease status and to evaluate the indicators’ accuracy. Youden's index was used for selecting the cutoff value. The area under the curve (AUC) was used as an estimation of diagnostic accuracy. In addition, sensitivity and specificity for Hb and Hct were obtained. Missing data from patients that were more than 50% were not included in the study. According to the analysis results, a p-value of 0.05 or below was accepted as statistically significant. For the sake of accuracy, the p-values are given with four digits following the decimal point.

## RESULTS

### Comparing baseline characteristics

Sociodemographic and clinical profiles and comparisons between groups are shown in Table 1.

**Table 1:** Sociodemographic and clinical characteristics of the patients diagnosed with polycythemia vera and secondary polycythemia.

		PV (n=85)	SP (n=115)	p-value
Age (years), median (IQR)		43 (18–84)	44 (18–79)	0.598*
Male sex—n (%)		75 (88.2)	106 (92.2)	0.348**
Smoking—n (%)		27 (31.8)	57 (49.6)	**0.012** **
Comorbid diseases				
	Diabetes (%)	10.6	21.7	**0.038** **
	Hypertension (%)	21.2	23.5	0.701**
	Dyslipidemia (%)	14.1	14.8	0.895**
	CAD (%)	5.9	7.8	0.595**
Hyperviscosity symptoms—(%)		45.9	22.6	**0.001** **
Eritromelalgia (%)		17.6	6.1	**0.010** **
Pruritis (%)		18.8	5.2	**0.002** **

Data are expressed as the median (IQR) or n (%). IQR: interquartile range; n: sample size; CAD: coronary artery disease;

* Mann-Whitney U test

** χ2 test. p<0.05 (in bold) indicates statistical significance.

### Laboratory findings and receiver operating characteristic analysis

Further statistical tests revealed significant differences between Hb and Hct results among the two groups in our study. If we now turn to other laboratory values (WBC, Neu, Plt, and LDH), they were also significantly higher in PV compared to the SP arm except for EPO (Table 2).

**Table 2:** Comparison of laboratory parameters between polycythemia vera and secondary polycythemia patients.

	PV (n=85)	SP (n=115)	p-value
Hb (g/dL)	17.9 (16.0–21.7)	17.4 (16.0–19.2)	**0.032** *
Hct (%)	54.1 (48.4–65.3)	52.0 (48.0–61.7)	**0.001** *
WBC (10^3^/μL)	8.4 (3.7–47.3)	7.4 (3.0–15.4)	**<0.001** *
LDH (mg/dL)	220 (139–1358)	209 (146–436)	**<0.001** *
Neutrophil (10^3^/μL)	5.3 (1.7–35.0)	4.3 (1.6–13.4)	**<0.001** *
Platelets (10^3^/μL)	271 (122–993)	241 (136–637)	**<0.001** *
Erythropoietin (IU/L)	6 (1–12)	9 (4–42)	**<0.001** *

All data are presented as the median (IQR); IQR: interquartile range; n: sample size; Hb: hemoglobin; Hct: hematocrit; WBC: white blood cell; LDH: lactate dehydrogenase

* Mann-Whitney U test. p<0.05 (in bold) indicates statistical significance.

**Figure 2:**
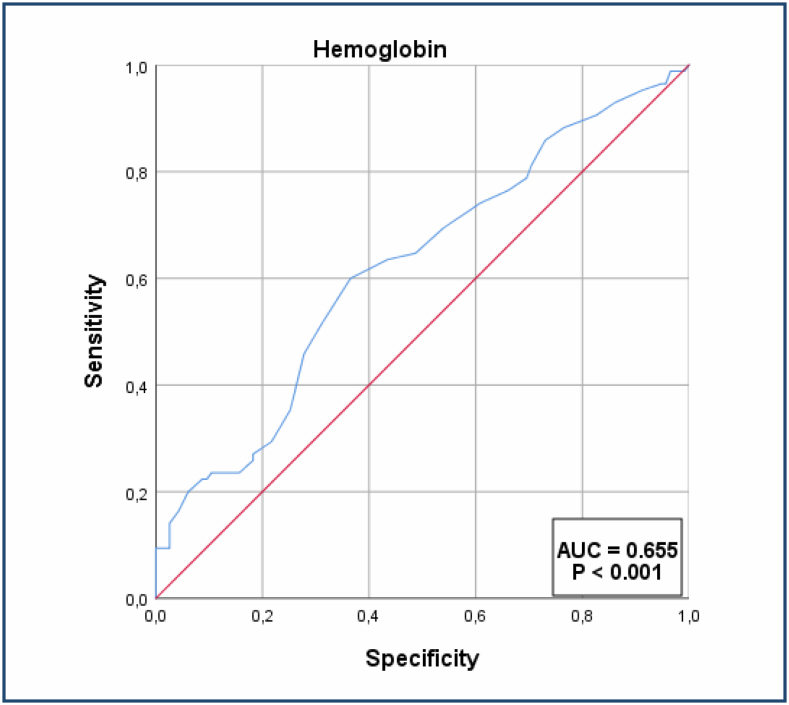
Receiver operating characteristic curve for the hemoglobin.

The distribution of Hb and Hct values of the patients in the study groups was recorded. The Hct value of 54.1% was predicted to diagnose PV with a sensitivity of 45% and a specificity of 80%. The area under the ROC curve (AUC) was 0.655 (95%CI 0.585–0.721) (Figure 2).

**Figure 3:**
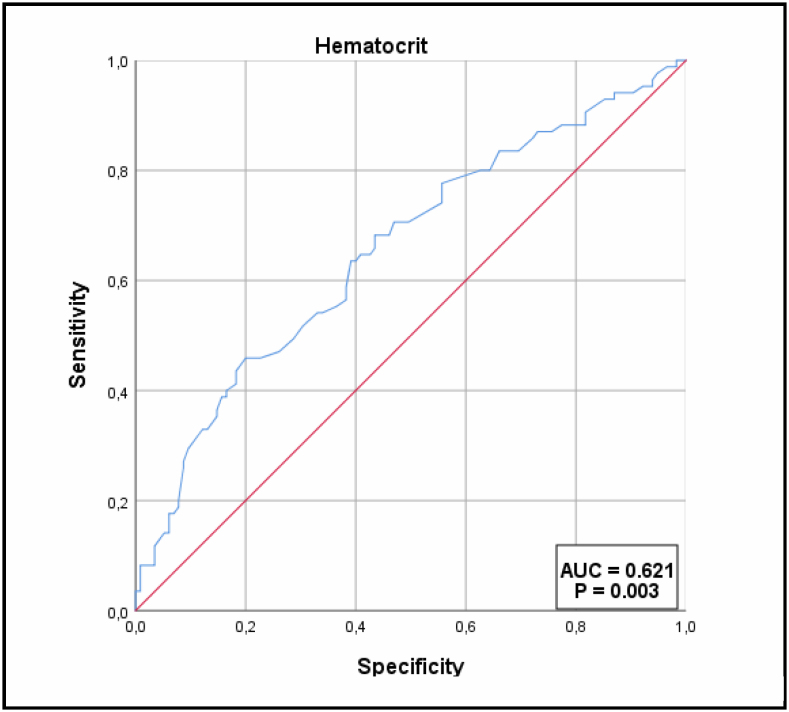
Receiver operating characteristic curve for the hematocrit.

Further analysis showed that an Hb value of 17.7 predicted the diagnosis of PV with a sensitivity of 60% and a specificity of 63%. The AUC was 0.621 (95%CI 0.550–0.689) (Figure 3).

## DISCUSSION

This study defined the factors related to the use of updated laboratory thresholds in 2016 and evaluated patients with polycythemia symptoms admitted to the hematology outpatient clinic. The present analysis was performed for two purposes. First, the study was to define the evaluation of PV and SP patients between the updated and old thresholds of Hb and HCT values^1^. Second, the study aimed to investigate the necessity of a bone marrow biopsy and genetic analysis to be used in further diagnosis.

The first focus point of discussion on the updated diagnostic criteria in 2016 is the lowering cutoff value in Hct and Hb for a major criterion^11^. A strong relationship between Hct and Hb levels and PV diagnosis has been debated in the literature^8,12-15^. In a study, it was suggested that bone marrow plays an important role in strengthening the relationship between Hb and Hct values and diagnosis, which can also help distinguish PV from other MPNs^16^. In a large-scale retrospective study based on these and similar concerns, 2016 WHO diagnostic criteria were strictly applied and performed on randomly selected people in the Canadian and Brazilian populations. The study was successfully predicted that the annual PV incidence would increase 12 times for men and 3 times for women when using 2016 criteria^17^. This study also suggested that standardizing diagnostic approaches for MPNs across the country could contribute to cost-effectiveness and avoid unnecessary further research. Barbui et al., conducted a study to investigate the usefulness of independent use as a major diagnostic criterion. The study also suggested that the Hb/Hct levels may lead to a significant increase in excessive diagnostic tests, such as EPO serum levels, molecular investigation (JAK2V617 and exon 12), and biopsies of the bone marrow^12^. It is also important to consider that 0.1% of all bone marrow biopsy interventions could cause serious side effects, including infection, bleeding, and even death^12,14^.

The most crucial aspect of the updated criteria in 2016 was to identify masked and clinically suspected cases^10,12^. Changing Hct and Hb thresholds in 2016 made some changes and was shown in a study to cause a 36% increase in the diagnosis of PV over the 2008 version^11^. In a study in which 2,056 suspected MPN patients were evaluated, 132 patients were diagnosed with the WHO 2008 criteria, while 154 patients were diagnosed with the WHO 2016 criteria^10^. The findings made an important contribution to the treatment of the patients who did not fully meet the diagnostic criteria. In contrast to earlier findings, when we considered the Hb and Hct thresholds of the updated criteria, none of the patients were diagnosed as PV instead of SP. One unanticipated finding was that the cutoff values found as 17.7 for Hb and 54.1 for Hct in the ROC analysis are above the current guideline^12^. A possible explanation for this might be geographic and demographic differences.

Another question in this study sought to determine the use of other complete blood count parameters for differential diagnosis. WBC, neutrophil, platelets, and LDH tests among the PV and SP groups were significantly different (p<0.001). This finding was also reported by Sandes et al., as mentioned in leukocytosis and thrombocytosis, which could be useful in PV diagnosis^14^. In cases with borderline levels of Hb and Hct, using other achievable laboratory parameters in combination for diagnosis may benefit researchers^12^.

Further statistical tests in the study revealed the prevalence of polycythemia was significantly higher in males compared to female patients. However, no significant difference between the two groups in gender was evident (p=0.348). These results are supported by many recent studies. In 426 PV cases followed between 2001 and 2011, the incidence rate was found to be lower in the female gender, especially with advanced age^18^. In contrast, in a systematic meta-analysis, the incidence of MPNs did not differ significantly between men and women^19^. Therefore, it should be noted that the updated diagnostic criteria for polycythemia should take into account the fundamental differences in hemoglobin and hematocrit between men and women, and gender differences observed in secondary polycythemia introduce the risk.

### Limitations

The limited number of patients and the low diagnostic efficacy of Hb and Hct values in this study can be counted among the limitations of the study. The study also partially illustrates the usefulness of ROC curve analysis applied to a common clinical laboratory test and how it can help in defining an appropriate range of values and a specific cutoff point for a particular population. Although the current study is based on a small sample of participants in PV, for the first time to the best of our knowledge, it adds to our understanding of the impact of the revised diagnostic criteria in 2016.

## CONCLUSION

As shown in our study, the updated criteria in the WHO classification applied to PV patients for differential diagnosis with high-normal Hb and Hct levels could cause unnecessary further investigation. Since Hb and Hct values used in the diagnostic criteria cannot be generalized, we think that a good clinical evaluation will prevent unnecessary investigations from being ordered in the diagnosis of PV. Differences in demographics and geography may force us to reassess the diagnostic criteria. A significant rise in needless tests for diagnosis, such as EPO serum dose, molecular testing (JAK2V617 and exon 12), and bone marrow biopsies, may result from the isolated use of the suggested Hb/Hct levels as a description of polycythemia. In spite of significant updates, we still need to support our findings with larger, prospective, and randomized studies to increase the diagnostic precision of MPNs.

## Data Availability

The data are available from the corresponding author upon reasonable request.
